# Cla4A, a Novel Regulator of Gene Expression Networks Required for Asexual and Insect-Pathogenic Lifecycles of *Beauveria bassiana*

**DOI:** 10.3390/ijms25126410

**Published:** 2024-06-10

**Authors:** Si-Yuan Xu, Rehab Abdelmonem Mohamed, Lei Yu, Sheng-Hua Ying, Ming-Guang Feng

**Affiliations:** Institute of Microbiology, College of Life Sciences, Zhejiang University, Hangzhou 310058, China

**Keywords:** P21-activated kinase, asexual development, stress response, fungal insect pathogenicity, transcriptional regulation, DNA-binding activity

## Abstract

Cla4, an orthologous p21-activated kinase crucial for non-entomopathogenic fungal lifestyles, has two paralogs (Cla4A/B) functionally unknown in hypocrealean entomopathogens. Here, we report a regulatory role of Cla4A in gene expression networks of *Beauveria bassiana* required for asexual and entomopathogenic lifecycles while Cla4B is functionally redundant. The deletion of *cla4A* resulted in severe growth defects, reduced stress tolerance, delayed conidiation, altered conidiation mode, impaired conidial quality, and abolished pathogenicity through cuticular penetration, contrasting with no phenotype affected by *cla4B* deletion. In ∆*cla4A*, 5288 dysregulated genes were associated with phenotypic defects, which were restored by targeted gene complementation. Among those, 3699 genes were downregulated, including more than 1300 abolished at the transcriptomic level. Hundreds of those downregulated genes were involved in the regulation of transcription, translation, and post-translational modifications and the organization and function of the nuclear chromosome, chromatin, and protein–DNA complex. DNA-binding elements in promoter regions of 130 dysregulated genes were predicted to be targeted by Cla4A domains. Samples of purified Cla4A extract were proven to bind promoter DNAs of 12 predicted genes involved in multiple stress-responsive pathways. Therefore, Cla4A acts as a novel regulator of genomic expression and stability and mediates gene expression networks required for insect-pathogenic fungal adaptations to the host and environment.

## 1. Introduction

*Beauveria bassiana*, an insect-pathogenic fungus with plant-endophytic and -saprophytic lifestyles, is considered to have evolved insect pathogenicity ~130 million years earlier than the *Metarhizium* lineage in Hypocreales and serves as a main source of wide-spectrum mycoinsecticides [1,2,3]. Molecular mechanisms underlying the fungal adaptation to the broadest host spectrum and diverse host habitats are increasingly revealed in the post-genomic era [4] but remain poorly understood.

Fungal adaptation to host and environment to a large extent is regulated by mitogen-activated protein kinase (MAPK) and calcium–calcineurin (C-C) signaling pathways [5,6]. MAPKs (Fus3/Kss1, Slt2, and Hog1), MAPK kinases (MAPKKs; Ste7, Pbs2, and Mkk1/2), and MAPK kinase kinases (MAPKKKs; Ste11, Ssk2/Ssk22, and Bck1/2) constitute the MAPK signaling cascades Fus3/Kss1-Ste7-Ste11, Mpk1/Slt2-Mkk1/2-Bck1/2, and Hog1-Pbs2-Ssk2/Ssk22 that mediate fungal responses to pheromones, cell wall integrity, high osmolarity, and other stresses [5]. In *Saccharomyces cerevisiae*, Ste11 acts as a MAPKKK in the Fus3/Kss1 cascade and one of two branches upstream of the Hog1 cascade, and it is activated by the p21-activated kinase Cla4 or Ste20 phosphorylated in response to the signal of pheromones, starving, or high osmolarity. The phosphorylation signal is passed through the MAPK cascades to activate downstream transcription factors (TFs) mediating gene expression networks. In early studies, Cla4 was revealed to mediate the cell cycle, polar growth, and cytokinesis by interacting with the guanine nucleotide exchange factor Cdc24 [6,7,8,9] and septin localization and assembly into the chitin ring essential for yeast budding and neck integrity [10,11,12]. In the Sho1 branch upstream of the Hog1 cascade, Cla4 and Ste20 are phosphorylated by binding to Cdc42 (small rho-like GTPase) and pass the phosphorylation signal through the cascade and downstream TFs [13]. The transduction of a pheromone signal by Cdc42 in the Fus3/Kss1 cascade relies on its interaction with Cla4 [14]. Additionally, Cla4 acts as a negative regulator of sterol biosynthesis [15,16] and orchestrates vacuole transmission from the mother to daughter cell [17,18]. Cla4 catalyzes histone H4T80 phosphorylation, which is crucial for DNA damage repair [19] and mediates glycerol biosynthesis [20]. The phosphorylation of its multiple sites is essential for the differentiation of yeast mating type and the transduction of pheromone signals in the Fus3 cascade [21]. These studies uncover a regulatory role of the yeast Cla4 in polar growth, cytokinesis, signal transduction, vacuolar inheritance, sterol/glycerol biosynthesis, histone phosphorylation, and DNA damage repair.

Either Cla4 or Ste20 is orthologous in yeast and filamentous fungal genomes analyzed previously [5]. Chm1, a Cla4 homolog of *Magnaporthe grisea* (=*Pyricularia grisea*), mediates conidiation and appressorium formation required for pathogenicity, contrasting with no role of Ste20/Mst20 in the mediation and null role of either Chm1 or Mst20 in activating the fungal MAPK cascade [22]. An interaction of Chm1 with Rac1 (small GTPase) is essential for *P. grisea* conidiation [23]. The role of Chm1 in appressorium formation has proven to be reliant on its collaboration with Cdc42, which is essential for septin assembly into the tip ring of a germ tube [24]. In *Ustilago maydis*, Cla4 triggers the degradation of the Rac1-Cdc24 complex required for polar growth [25,26] and regulates filamentous response to low nitrogen signal [27]. The disruption of *cla4* in *Bipolaris maydis* led to severe defects in hyphal growth, sporulation, and pathogenicity, suggesting a role of Cla4 in the fungal lifecycle in vitro and in vivo [28]. Cla4 S685 is a conserved phosphorylation site critical for the sexual development of *Sordaria macrospora* [29]. In addition, Cla4 modulates hyphal septation and asexual development in *Aspergillus fumigatus* [30]. Previous studies demonstrate differential or different functions of Cla4 homologs in fungi with different lifestyles. However, little attention has been paid to linkages of Cla4-modulated gene expression networks with yeast or non-yeast fungal lifecycles.

The stress-responsive pathways of *B. bassiana* have been studied in the past decade. The Mpk1/Slt2 cascade mediates responses to cell wall-perturbing, hyperosmotic, fungicidal, thermal, and UV stresses through a signaling interplay with the Hog1 cascade [31]. The unbranched Hog1 cascade regulates cell tolerance to oxidation, fungicides, and heat shock through the transduction of phosphorylation signals and the orchestration of fungal conidiation and virulence, contrasting with Ste11, which is proven to act as a MAPKKK only in the Fus3 cascade that modulates hyphal growth, asexual development, and insect pathogenicity [4,32]. Most components of the C-C pathway play important, but differential, roles in the fungal lifecycle in vitro and in vivo, including three calcineurin subunits (CnA1, CnA2, and CnB), six P-type Ca^2+^-ATPases (Pmr1, Eca1, Spf1, and PmcA–C), three proline rotamases (Fpr1–3), and the TF Crz1 [33,34,35]. Compared to the nearly full coverage of the C-C pathway, previous studies have focused mainly on the characterization of three kinases in each MAPK cascade but very limitedly on upstream signaling factors or downstream TFs in *B. bassiana*. Little is known about the transcriptional linkage of an upstream factor with multiple proteins/enzymes and TFs in the pathways.

As an ancient insect pathogen, *B. bassiana* have seemingly evolved some special signaling factors upstream of the MAPK cascades. For example, the fungus has nine single WSC (cell wall stress-responsive component) domain-containing proteins (Wsc1A–I) with transmembrane activities [36], contrasting with no more than four WSC proteins (Wsc1–4) that are considered to perceive external stress cues upstream of the Mpk1/Slt2 cascade in model fungi [5]. Of those, Wsc1I acts as a sensor of hyperosmotic, oxidative, and cell wall-perturbing stress cues and can pass phosphorylation signals of those cues through the Hog1 cascade [37], providing an insight into its crucial role in the signaling/functional interplay of the Mpk1/Slt2 and Hog1 cascades revealed previously [31]. In a survey of fungal genomes, we found two functionally unknown Cla4 paralogs (Cla4A/B) in hypocrealean insect pathogens including *B. bassiana* as well as an orthologous Cla4 in the yeast and filamentous fungi examined previously [5]. This study sought to elucidate their biological functions in *B. bassiana* through analyses of targeted gene deletion mutants and control (wild-type and complemented) strains. Results demonstrate an essential role of Cla4A in radial growth, conidiation, stress response, and insect pathogenicity, as well as the functional redundancy of Cla4B, in *B. bassiana*. Importantly, Cla4A was found to target promotor DNA-binding elements of many genes across pathways and regulate genome-wide gene expression networks required for the fungal lifecycle.

## 2. Results

### 2.1. Domain Architecture of Fungal Cla4 Homologs

**Figure 1:**
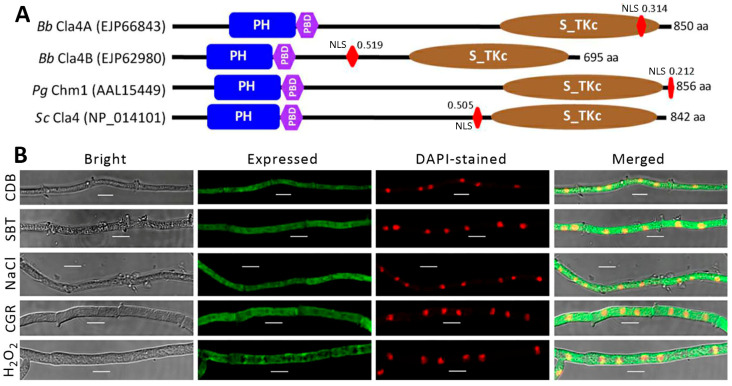
Features of Cla4 paralogs in *B. bassiana* (*Bb*). (**A**) Comparative domain architectures of Cla4 homologs in *B. bassiana*, *P. grisea* (*Pg*), and *S. cerevisiae* (*Sc*). (**B**) LSCM images (scale: 5 μm) for subcellular localization of Cla4A-GFP expressed in *Bb*. Observed hyphae were prepared by 2 h shaking incubation in CDB alone (control) or containing sorbitol (1 M), NaCl (0.4 M), Congo red (3 μg/mL), or H_2_O_2_ (2 mM) after 60 h submerged incubation of conidia in SDBY at 25 °C and staining with the nuclear dye DAPI.

Cla4 homologs in entomopathogenic and non-entomopathogenic ascomycetes were located by search through NCBI databases using the query of *P. grisea* Chm1 (AAL15449, 856 amino acids (aa)) or *S. cerevisiae* Cla4 (NP_014101, 842 aa) and clustered to phylogenetic clades associated with fungal lineages (Figure S1). While orthologous Cla4 exists in the non-entomopathogenic lineages examined, Cla4A and Cla4B were found in all hypocrealean entomopathogens except *Moelleriella libera*, which harbors Cla4 ortholog-like *Ascosphaera apis* (Onygenales), *Sporothrix insectorum* (Ophiostomatales), and non-entomopathogenic ascomycetes. The *B. bassiana* Cla4A had higher sequence identity to Cla4A and Cla4B homologs of Cordycipitaceae (90–99% and 51–64%) than of Clavicipitaceae (68–76% and 45–64%). In *B. bassiana*, Cla4A (EJP66843, 850 aa) and Cla4B (EJP62980, 695 aa) were featured with the same conserved PH (Pleckstrin Homology), PBD (p21-Rho-binding), and S_TKc (Serine/Threonine protein kinases, catalytic) domains as either query (Figure 1A).

The two paralogs showed sequence identities of 74.5% (total score 727, coverage 59%, e-value zero) and 55.1% (total score 647, coverage 65%, e-value 8 × 10^−137^) to the *P. grisea* query and 35.6% (total score 128, coverage 21%, e-value 9 × 10^−31^) and 45.3% (total score 452, coverage 71%, e-value 3 × 10^−102^) to the yeast query. A nuclear localization signal (NLS) motif was also predicted from the mentioned paralogs and queries at maximal probabilities of 0.21–0.52. These data demonstrate a conserved domain architecture for Cla4 homologs in the yeast and filamentous fungi.

### 2.2. Subcellular Localization of Cla4A in B. bassiana

A transgenic strain expressing the fusion protein Cla4A-GFP in a wild-type strain (*B. bassiana* ARSEF2860, designated WT) was analyzed to reveal its subcellular localization. Laser scanning confocal microscopic (LSCM) images showed the consistent localization of Cla4A-GFP in both cytoplasm and nuclei of hyphae stained with a nucleus-specific dye after 2 h of shaking incubation in Czapek–Dox broth (CDB) alone or containing sorbitol, NaCl, Congo red, or H_2_O_2_ (Figure 1B). These images indicated a nucleocytoplasmic localization of Cla4A in the absence or presence of a stress cue.

### 2.3. Comparative Roles of Cla4A and Cla4B in Asexual Lifecycle

**Figure 2:**
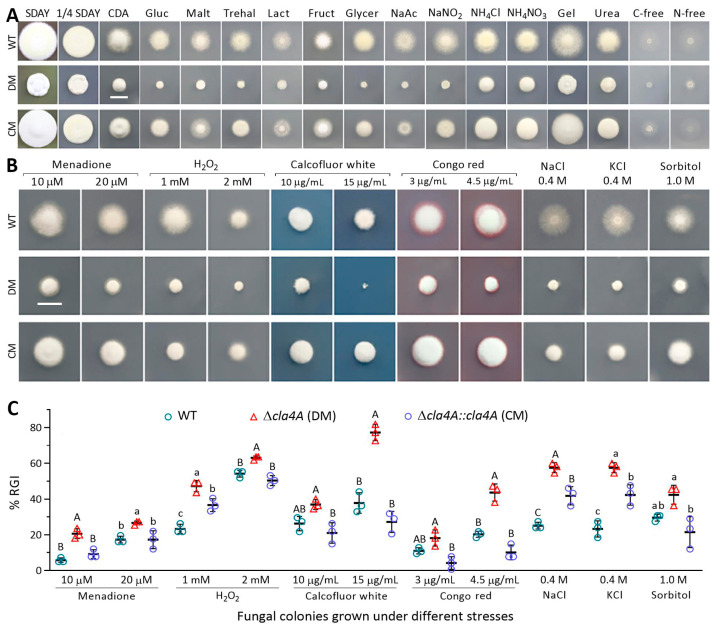
Impact of *cla4A* deletion on radial growth and stress response of *B. bassiana*. (**A**) Images (scale: 10 mm) of fungal colonies incubated at 25 °C and 12:12 (L–D) for 7 days on SDAY, 1/4 SDAY, CDA, and CDAs amended with different carbon or nitrogen sources. (**B**,**C**) Images (scale: 10 mm) and relative growth inhibition (RGI) percentages of fungal colonies incubated at the optimal regime for 7 days on CDA containing indicated chemical stressors. Each colony was initiated with ~10^3^ conidia. Different uppercase or lowercase letters denote significant differences at *p* < 0.01 or 0.05 (Tukey’s test). Error bard: standard deviations (SDs) from three independent replicates.

The mutants of *cla4A* and *cla4B* were created in the WT background and identified via PCR and real-time quantitative PCR (qPCR) analyses (Figure S2) with paired primers (Table S1). The deletion of *cla4A* resulted in severe growth defects on Sabouraud dextrose agar plus yeast extract (SDAY), 1/4 SDAY (1/4 nutrition strength of SDAY), CDA (agar-inclusive CDB), and CDAs amended with different carbon/nitrogen sources (Figure 2A). Compared with control strains, the ∆*cla4A* mutant was significantly more sensitive to oxidation, cell wall perturbation, and high osmolarity and exhibited hypersensitivity to calcofluor white (15 μg/mL), Congo red (4.5 μg/mL), and NaCl or KCl (0.4 M) (Figure 2B,C).

**Figure 3:**
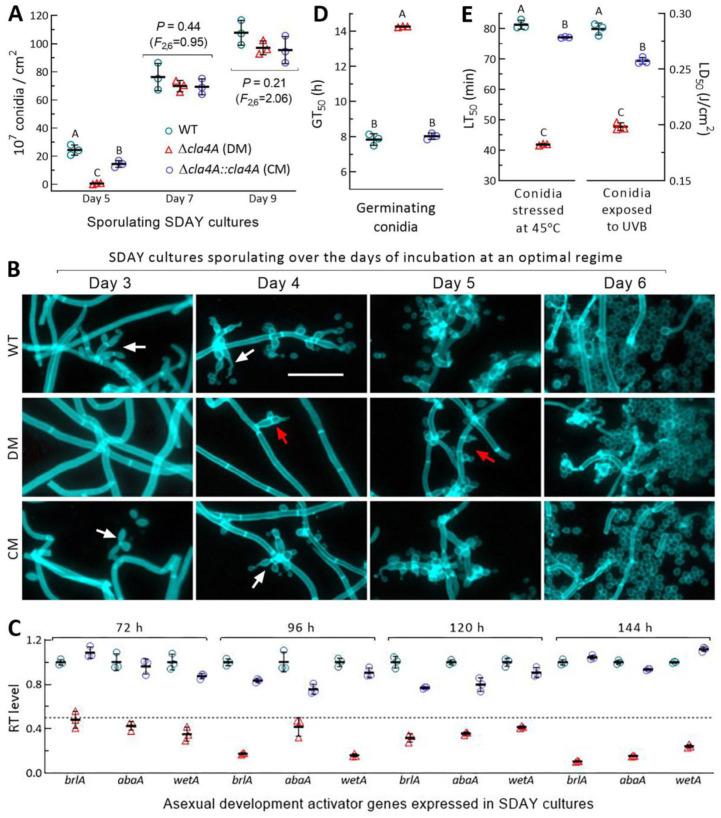
Impact of *cla4A* deletion on conidiation and conidial quality of *B. bassiana*. (**A**) Conidial yields measured from the 5-, 7-, and 9-day-old cultures initiated by spreading 100 μL of conidial suspension (10^7^ conidia/mL) on SDAY plates at 25 °C and 12:12 (L–D). (**B**,**C**) Microscopic images (scale: 20 μm) of conidiation status and relative transcript (RT) levels of three development activator genes in the 3- to 6-day-old SDAY cultures. Culture samples were stained with calcofluor white. White and red arrows indicate normal zigzag rachises and abnormal conidiating cells, respectively. The dashed line denotes a significance of one-fold downregulation. (**D**,**E**) Quality indices of conidia from the 9-day-old SDAY cultures. Different uppercase letters denote a significance of *p* < 0.01 (Tukey’s test). Error bars: SDs from three independent replicates.

As a core trait of the asexual cycle, conidiation capacity was assessed during a 9-day incubation at the optimal regime on SDAY plates spread with 100 μL aliquots of conidial suspension. Conidial yields (Figure 3A) were sharply reduced by 98% in the 5-day-old SDAY cultures of ∆*cla4A* versus WT but showed little variation among the tested strains on day 7 (*F*_2,6_ = 0.95, *p* = 0.44) or 9 (*F*_2,6_ = 2.06, *p* = 0.21). The control strains were observed forming zigzag rachises (special conidiophores) to initiate conidiation on day 3, followed by rapid increases of zigzag rachises and conidiation levels on days 4 and 5 (Figure 3B). In contrast, the ∆*cla4A* hyphae were not differentiated on day 3 but observed to start the formation of specialized cells on day 4. Such cells became abundant on day 5 and appeared to form singular conidia by yeast-like budding at the tip, making them distinct from the clustered zigzag rachises to form spore balls that increase rapidly in size and density and self-scatter into conidial powder upon maturation [38]. In qPCR analysis, three developmental activator genes (*brlA*, *abaA,* and *wetA*) required for asexual development [39,40] were significantly downregulated in the 3- to 6-day-old ∆*cla4A* cultures (Figure 3C), suggesting a blockage of normal conidiation. Despite a delay, surprisingly, the abnormal conidiation appeared to proceed rapidly, leading to high-level conidiation on day 6 and no difference in the conidial yield measured afterwards. However, the ∆*cla4A* mutant was severely compromised in conidial quality, including 82% reduction in viability (shown with median germination time (GT_50_) at 25 °C, Figure 3D), 49% decrease in heat (45 °C) tolerance (median lethal time (LT_50_), left in Figure 3E), and 31% drop in UVB resistance (median lethal dose (LD_50_), right in Figure 3E).

The deletion of *cla4B* caused no changes in phenotypes associated with the fungal asexual cycle (Figure S3A–C). These data indicated an essentiality of Cla4A but a dispensability of Cla4B for hyphal growth, asexual development, and cellular responses to external stresses. Particularly, Cla4A acted as a negative regulator of the abnormal, but highly efficient, conidiation in *B. bassiana*.

### 2.4. Comparative Roles of Cla4A and Cla4B in Insect-Pathogenic Lifecycle

**Figure 4:**
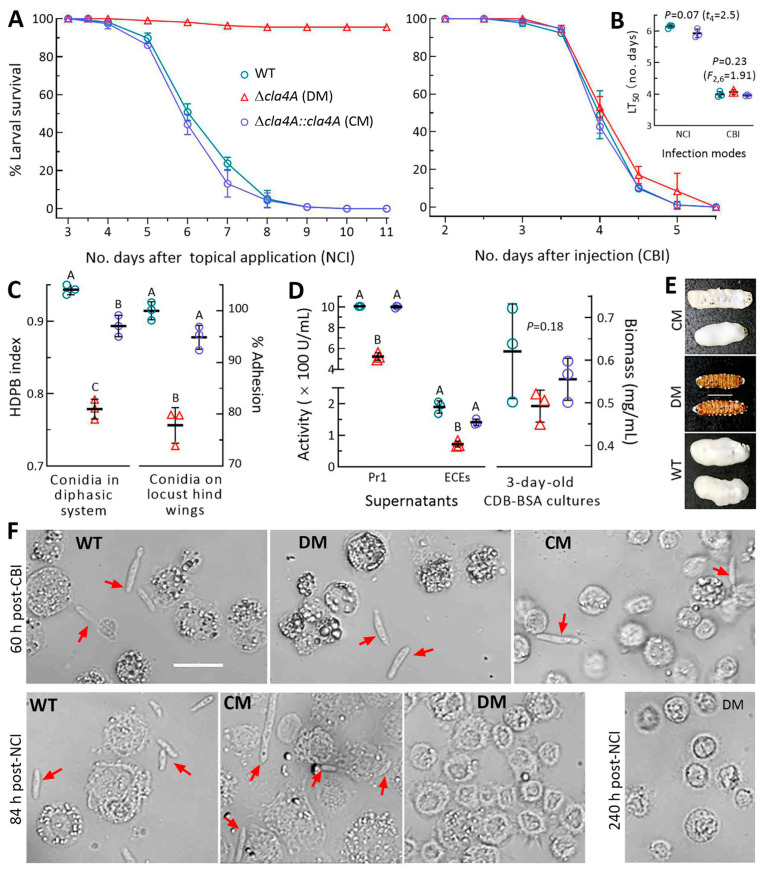
Essentiality of *cla4A* for insect pathogenicity of *B. bassiana* through cuticular penetration. (**A**,**B**) Percent survival trends of *G. mellonella* larvae after normal cuticle infection (NCI) and cuticle-bypassing infection (CBI) and LT_50_ estimates made by the modeling analysis of each trend. Note that LT_50_ was not assessable for DM via NCI. (**C**) Conidial hydrophobicity (HDPB) and percent adhesion assessed in a diphasic (aqueous–organic) system and a locust hindwing assay, respectively. (**D**) Total activities of cuticle-degrading ECEs and Pr1 proteases assessed in the supernatants of 3-day-old CDB-BSA cultures and biomass levels in the cultures. (**E**) Images (scale: 10 mm) of fungal outgrowths on the surfaces of cadavers 5 days post-death from CBI. (**F**) Microscopic images (scale: 20 μm) of hemolymph samples taken from surviving larvae after NCI and CBI. Red arrows indicate hyphal bodies proliferating via yeast-like budding. Note that DM formed no hyphal body 84 and 240 h post-NCI and lost an ability to penetrate through cadaver cuticle for outgrowth. Different uppercase letters in each treatment denote a significance of *p* < 0.01 (Tukey’s test). Error bar: SD from three independent replicates.

In standard bioassays, control strains killed all *Galleria mellonella* larvae (last instar) within 9 days after the topical application (immersion) of a 10^7^ conidia/mL suspension for normal cuticle infection (NCI) and within 5 days after the intrahemocoel injection of ~500 conidia per larva for cuticle-bypassing infection (CBI) (Figure 4A). The ∆*cla4A* mutant killed all larvae via CBI, as did control strains, but was unable to kill the model insect via NCI. This resulted in an LT_50_ that was the same as those of control strains via CBI (*F*_2,6_ = 1.91, *p* = 0.23) but not assessable via NCI (Figure 4B). Neither NCI nor CBI was affected in the absence of *cla4B* (Figure S3D,E).

The abolished ∆*cla4A* pathogenicity via NCI correlated with reductions in conidial hydrophobicity (18%) and cuticle adhesion (22%) that were required for initial NCI (Figure 4C) and correlated with decreased activities of cuticle-degrading extracellular (proteolytic, chitinolytic and lipolytic) enzymes (ECEs, 62%) and Pr1 family proteases (48%, Figure 4D) that were vital for successful NCI [41,42]. Since unicellular hyphal bodies proliferating via yeast-like budding in insect hemocoel become septate hyphae to penetrate the cuticle for outgrowth upon host death, the larvae killed via CBI were held at 25 °C for fungal outgrowth to show the tested strains’ abilities to penetrate through insect cuticle. Consequently, the control strains formed a heavy layer of outgrowths covering all cadaver surfaces 5 days post-death (Figure 4E). In contrast, the ∆*cla4A* mutant’s outgrowth was strictly limited to spiracles, mouthparts, and anuses of cadavers so that almost all cadaver surfaces were bald. The microscopic examination of hemolymph samples taken from surviving larvae revealed that the ∆*cla4A* mutant formed abundant hypha bodies 60 h post-CBI like the control strains but no hyphal body 84 and 240 h post-NCI (Figure 4F).

Altogether, Cla4A was indispensable for the fungal insect-pathogenic lifecycle. The indispensability depended on its role in cellular processes and events required for NCI, including mainly conidial hydrophobicity/adhesion and the secretion of cuticle-degrading enzymes. However, Cla4B played no role in the fungal lifecycle in vivo.

### 2.5. Profound Effect of Cla4A on Genomic Expression and Stability

**Figure 5:**
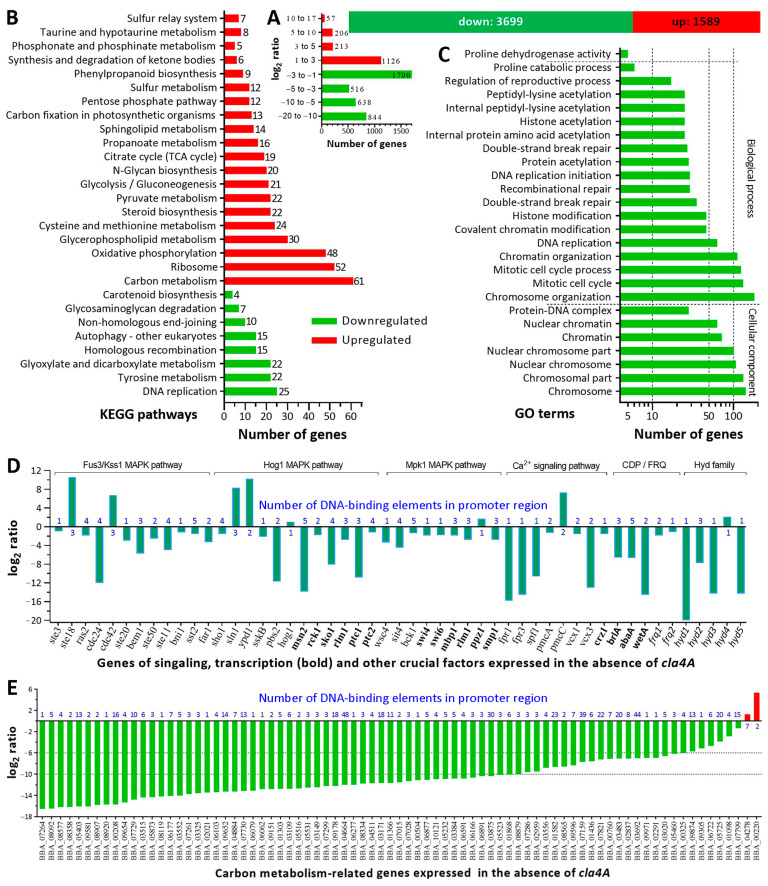
Profound effect of *cla4A* deletion on genomic expression and stability of *B. bassiana*. (**A**) Counts of DEGs identified from the transcriptome of ∆*cla4A* versus WT. (**B**,**C**) Counts of DEGs significantly enriched (*p* < 0.05) to KEGG pathways and GO terms (see Table S4 for full insight), respectively. (**D**,**E**) The log_2_ ratios of 41 DEGs in stress-responsive MAPK and C-C pathways, 10 DEGs required for asexual development and cell hydrophobicity, and 79 DEGs presumably involved in carbon metabolism. Blue value marked on each DEG denotes the number of its promoter DNA-binding elements predicted to be targeted by Cla4A domain(s).

In the transcriptomic analysis of ∆*cla4A* versus WT, 5288 differentially expressed genes (DEGs, up/down ratio 1589:3699; the same applies to the ratios below) were identified at significant levels of at least one-fold change and FDR (false discovery rate) < 0.05 (Figure 5A, Table S2), taking 51% of the fungal genome [43]. More than 1300 genes showed log_2_ ratios ≤ –6, indicating that their expressions were virtually abolished in ∆*cla4A*.

Kyoto Encyclopedia of Genes and Genomes (KEGG) analysis resulted in 541 DEGs (421:120) enriched significantly to 8 downregulated and 20 upregulated pathways (Figure 5B, Table S3). Among the downregulated pathways, 3 were involved in DNA replication and repair; the remaining were involved in carbohydrate metabolism, amino acid metabolism, transport and catabolism, glycan biosynthesis and metabolism, and the metabolism of terpenoids and polyketides, respectively. The upregulated pathways were involved mainly in carbon metabolism, ribosome, oxidative phosphorylation, glycerophospholipid metabolism, cysteine and methionine metabolism, steroid biosynthesis, glycolysis/gluconeogenesis, pyruvate metabolism, N-glycan biosynthesis, citrate cycle, propanoate metabolism, sphingolipid metabolism, carbon fixation, pentose phosphate pathway, and sulfur metabolism. Gene Ontology (GO) analysis resulted in enrichments of 1622 genes to 26 downregulated GO terms (Figure 5C) and of many more to 303 upregulated GO terms (Table S4). A total of 7 downregulated GO terms fell in cellular component, including chromosome, chromosomal part, nuclear chromosome, nuclear chromosome part, chromatin, nuclear chromatin, and protein–DNA complex. Classified to biological process were 18 downregulated GO terms, most of which were involved in chromosome organization, mitotic cell cycle, chromatin organization, covalent chromatin modification, histone modification, protein acetylation, histone acetylation, and DNA replication and repair. The downregulated KEGG pathways and GO terms demonstrated an indispensability of Cla4A for fungal genomic expression and stability.

Listed in Table S5 were 229 DEGs (76:153) encoding various TFs, 98 (9:89) involved in reverse transcription and translation, 141 (56:85) involved in posttranslational modifications, 36 (7:29) involved in DNA replication, recombination and repair, 113 (18:95) involved in ribosome, proteasome and chromatin remodeling, 281 (117:164) involved in cellular transport, homeostasis and multidrug resistance, and 95 (22:73) involved in signal transduction. Among those involved in post-translational modifications and chromatin remodeling, 24 DEGs (2:22) were the coding genes of histones (H2A, H2A.Z, H2B, H3, H4, H4.1 and H5) and histone acetyltransferases, deacetylases, and chaperones. Also listed in Table S5 are 22 DEGs (5:17) involved in cytoskeleton, 14 (3:11) involved in cell cycle and division, 9 (0:9) involved in autophagy, and 37 (15:22) involved in mitochondrial structure and function. Importantly, many DEGs were associated with phenotypic defects of ∆*cla4A*, including 44 (12:32) involved in fungal entomopathogenicity, 138 (52:86) involved in antioxidant response, and 26 and 44 (12:14 and 17:27) involved in responses to heat shock and cell wall perturbation, respectively.

Shown in Figure 5D are 41 DEGs (6:35) encoding signaling factors and TFs in the MAPK and C-C pathways [5] and another 10 important DEGs (1:9), which encode three asexual developmental activators [39,40], two FRQ proteins (Frq1/2) essential for non-rhythmic conidiation [44], and five hydrophobins including Hyd1/2 determinant to conidial hydrophobicity and adhesion in *B. bassiana* [45]. To reveal a regulatory role of Cla4A across the pathways, its domains (Residues 100–209, 212–247 and/or 562–830) were predicted to target 1–5 DNA-binding elements in the promoter regions of those DEGs (blue values in Figure 5D). Further, 1–48 DNA-binding elements in the promoter regions of another 79 DEGs (2:77) presumably involved in carbon metabolism were also predicted as target sites of Cla4A domains (Figure 5E). The predicted target sites hint that Cla4A may act as a regulator of gene expression networks in *B. bassiana*.

### 2.6. Validating DNA-Binding Activity of Cla4A

**Figure 6:**
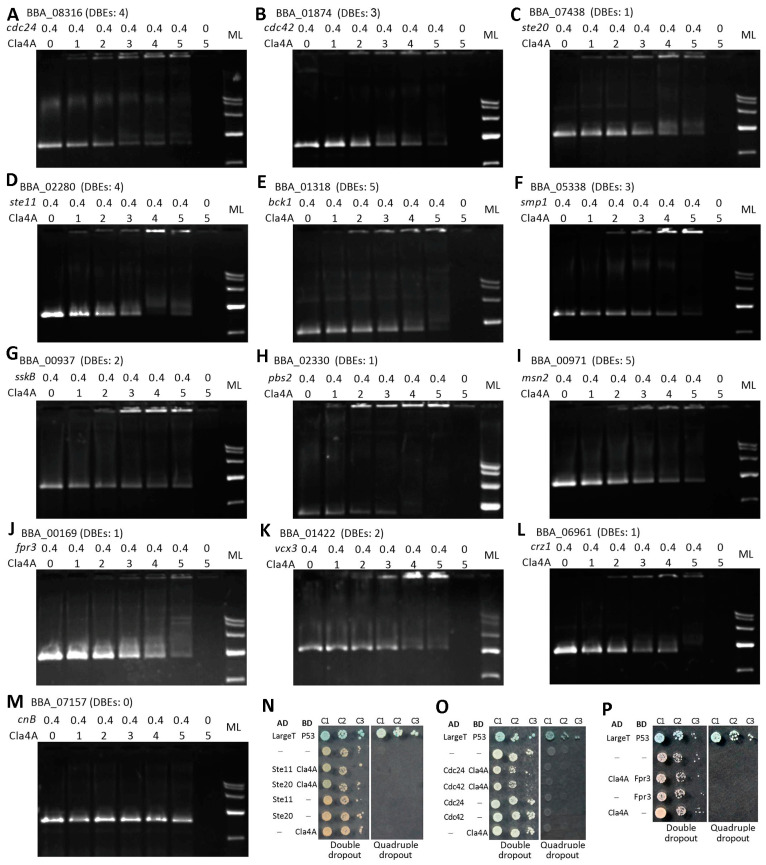
(**A**–**L**) EMSAs for the activities of purified Cla4A extract binding to the promoter DNAs of 12 genes dysregulated in the absence of *cla4A*. The tag locus of each tested gene is followed by the number of its promoter DNA-binding elements (DBEs) predicted to be targeted by Cla4A domains. In each EMSA, 0.4 μg DNA samples were mixed with 1, 2, 3, 4, and 5 μg of Cla4A extract in 10 μL aliquots of binding buffer for a 30 min reaction, followed by agarose gel electrophoresis to show Cla4A-bound DNA signals (top) and weakening DNA signals (bottom). Internal negative control was 10 μL binding buffer containing 0.4 μg DNA or 5 μg protein alone. (**M**) The negative control of EMSA for the activity of Cla4A extract binding to the *cnB* promoter DNA with no Cla4A-targeting site. (**N**–**P**) Y2H assays for interactions of Cla4A with Ste11 and Ste20, Cdc24 and Cdc42, and Fpr3, respectively. All yeast colonies were initiated with 5 × 10^4^ (C1), 5 × 10^3^ (C2) and 5 × 10^2^ (C3) cells and incubated for 3 days at 30 °C.

The predicted DNA-binding activity of Cla4A was verified in electrophoretic mobility shift assays (EMSAs) performed to detect whether 1–5 μg samples of purified Cla4A extract (93.24 kDa) bind 0.4 μg promoter DNA samples of 12 DEGs as Cla4A-targeting genes that function upstream or downstream of the MAPK and C-C pathways [7]. In each EMSA, lower DNA signal weakened with increasing protein sample sizes, while the upper protein signal was increasingly intensified with weakening DNA signal, but no signal was detected in the lane uploaded with DNA or protein samples alone (Figure 6A–L). In contrast, protein signal was absent in the lanes uploaded with both the protein samples and the DNA samples of *cnB* promoter lacking a predictable Cla4A-targeting site (negative control), leading to the DNA signal being unaffected in the lanes (Figure 6M). These results confirmed the activity of Cla4A binding to each of the tested promoter DNAs comprising 1–5 Cla4A-targeting sites.

Next, yeast two-hybrid (Y2H) assays were carried out to detect whether Cla4A is involved in possible interactions, such as the yeast Cla4-Cdc42 interaction [7,14]. Results revealed no interaction of Cla4A with Ste11, Ste20, Cdc24, Cdc42, or Fpr3 (Figure 6N–P), excluding post-translational links of Cla4A to the tested enzymes, although it targets their coding genes.

## 3. Discussion

Cla4 has proven to be orthologous in non-entomopathogenic ascomycetes as documented [5] but evolved into two paralogs in all hypocrealean entomopathogens except *M. libera*. Our study uncovers the functional redundancy of Cla4B but an essentiality of Cla4A for the in vitro and in vivo lifecycles and the genomic expression and stability of *B. bassiana*, as discussed below.

The essentiality of Cla4A for asexual and entomopathogenic lifecycles is revealed by the ∆*cla4A* mutant compromised in radial growth, responses to multiple stresses, conidiation, and conidial quality and abolished in pathogenicity. The abolished pathogenicity correlates well with decreases in conidial viability, hydrophobicity, adhesion, and total activities of secreted cuticle-degrading enzymes required for NCI [41,42,45]. The altered phenotypes are due to the malfunction of gene clusters in ∆*cla4A*, such as those involved in host infection, virulence, and other related cellular processes. Noticeably, these compromised phenotypes also appeared in the null mutants of *ste11*, *sskB*, *pbs2*, *bck1*, *frp3*, *msn2,* and *crz1* [4,32,33,35,46], which function in the MAPK and C-C pathways [5] but were downregulated in ∆*cla4A*. Previous ∆*cla4*/∆*chm1*mutants in *A. nidulans* and *P. grisea* were compromised in asexual development and pathogenicity [22,30]. Their phenotypic defects are subtly different from those displayed by the present ∆*cla4A* mutant. For example, it lost all ability to penetrate through insect cuticle but retained a capability of hemocoel colonization. The delay in its abnormal conidiation concurred with the consistent downregulation of *brlA*, *abaA,* and *wetA* required for normal conidiation and conidial maturation [39,40] but had no impact on conidiation capacity. The downregulation implies that Cla4A may act as a positive regulator of the three developmental activators and a switch to the abnormal conidiation when they were collectively downregulated to block normal conidiation. However, mechanisms underlying the abnormal conidiation that affected conidial quality rather than conidiation capacity remain unclear in *B. bassiana*.

The pleiotropic effect of Cla4A coincides with its regulatory role in gene expression networks. The Cla4A domains are structurally different from DNA-binding domains of typical TFs but were predicted to target promoter DNA-binding elements of 130 genes, which function in various pathways and were markedly dysregulated in ∆*cla4A*. Importantly, subsequent EMSAs confirmed that the promoter DNAs of 12 genes predicted to be targeted by Cla4A domains were bound to purified Cla4A samples. Both the dysregulation of those Cla4A-targeting genes and the validation of its DNA-binding activity indicate that Cla4A acts as a core regulator of gene expression networks across important pathways in *B. bassiana*. However, Cla4A was revealed to not interact with Ste11, Ste20, Cdc24, or Cdc42 upstream of the Fus3 cascade and Fpr3 in the C-C pathway, although their coding genes were proven targets of Cla4A. This is distinct from a Cla4-Cdc42 interaction involved in the yeast cell cycle, polar growth, cytokinesis, and pheromone signal transduction [7,8,9,14] and a collaboration of Chm1 with Cdc42 in appressorium formation essential for the pathogenicity of *P. grisea* [24].

The genome-wide regulatory role of Cla4A in *B. bassiana* is revealed by the dysregulation of many genes involved in genomic stability. More than 1300 genes were not expressed in ∆*cla4A*. Many of them are involved in the mediation of transcription, reverse transcription, translation, and post-translational modifications that function as fundamental mechanisms of chromatin remodeling and transcriptional mediation [47]. Particularly, almost all histones and multiple histone acetyltransferases, deacetylases, and chaperones crucial for genomic stability [48,49] were collectively downregulated, leading to the impairment, disorganization, and/or malfunction of nuclear chromosome, chromatin, and protein–DNA complex and the dysregulation of 51% of genes in the ∆*cla4A* genome. Due to the validated DNA-binding activity, Cla4A appears to target the coding genes of the TFs and crucial proteins or enzymes that were transcriptionally abolished or sharply repressed in ∆*cla4A*. We infer that Cla4A directly mediates the expression of its target genes and indirectly mediates the expression of many more genes to be regulated by its targets. Therefore, Cla4A exerts a profound and comprehensive effect on the genomic expression and stability of *B. bassiana*.

In conclusion, Cla4A acts as a novel regulator of gene expression networks required for the asexual and insect-pathogenic lifecycles of *B. bassiana*. Cla4B appears to be functionally redundant. This finding unveils a significance of Cla4A for fungal insect-pathogenic lifestyle.

## 4. Materials and Methods

### 4.1. Recognition and Sequence Analysis of Fungal Cla4A Homologs

Yeast Cla4 and *P. grisea* Chm1 were used as queries to search through the NCBI databases of entomopathogenic and non-entomopathogenic ascomycetes at https://blast.ncbi.nlm.nih.gov/blast.cgi (accessed on 6 April 2024). All located homologs were phylogenetically clustered with a maximum likelihood method in MEGA11 (https://www.megasoftware.net/) (accessed on 6 April 2024). Conserved domains and NLS motifs were predicted from the amino acid sequences of two *B. bassiana* paralogs and two queries at https://smart.embl-heidelberg.de/ (accessed on 6 April 2024) and https://www.novopro.cn/tools/nls-signal-prediction (accessed on 6 April 2024), respectively.

### 4.2. Generation of cla4A and cla4B Mutants

The full-length coding sequence plus partial flank regions of *cla4A* (2944 bp) or *cla4B* (2393 bp) was deleted from the WT genome (Figure S2A,B) by homologous recombination of the vector p0380-5′*x*-bar-3′*x* (*x*, *cla4A* or *cla4B*) via *Agrobacterium*-mediated transformation. Targeted gene complementation was achieved via the ectopic integration of p0380-*x*-sur into an identified ∆*x* mutant. Putative mutant colonies were screened with the resistance of *bar* to phosphinothricin (200 μg/mL) or the resistance of *sur* to chlorimuron ethyl (10 μg/mL). The homologous recombination events were revealed through the PCR detection of targeted DNA fragments (Figure S2C,D) and validated with the expression levels of either target gene in the cDNA samples of its mutants versus WT analyzed through qPCR (Figure S2E,F). Primers used for vector construction and targeted gene detection are listed in Table S1.

### 4.3. Phenotypic Experiments

Experiments comprising three independent replicates were carried out to assess fungal lifecycle-related phenotypes in the presence/absence of *cla4A* or *cla4B*. The radial growth of each strain was initiated by spotting ~10^3^ conidia (in 1 μL suspension of 10^6^ conidia/mL) on the plates of various media (Figure 2A) and of CDA alone (control) or supplemented with different chemical stressors (Figure 2B). After 7 days of incubation at 25 °C and 12:12 (L–D), fungal colonies were photographed, and then their diameters were measured. Relative growth inhibition (%) was computed as an index of each strain’s sensitivity to a given stress using the diameters of control and stressed colonies [32].

To assess conidiation capacity, 100 μL aliquots of conidial suspension (10^7^ conidia/mL) were spread on SDAY plates (9 cm diameter) and incubated for 9 days at 25 °C and 12:12 (L–D). Conidial yield in each of the 5-, 7-, and 9-day-old cultures was measured as the number of conidia per square centimeter [32]. Culture samples of the 3- to 6-day-old cultures were stained with calcofluor white and microscopically examined for conidiation status. Conidia from the 9-day-old cultures were used to assess quality indices, including GT_50_ (h) as a viability index, LT_50_ (min) as an index of tolerance to wet-heat stress at 45 °C, and LD_50_ (J/cm^2^) as an index of resistance to UVB irradiation [34]. Conidial hydrophobicity and adhesion to insect cuticle were assessed in diphasic (aqueous–organic) and locust hindwing assays [45,50], respectively.

The virulence of each strain against *G. mellonella* larvae was assayed via NCI and CBI. NCI was initiated in three groups of ~35 larvae immersed for 10 s in 40 mL aliquots of a 10^7^ conidia/mL suspension. CBI was initiated by injecting 5 μL of a 10^5^ conidia/mL suspension into the hemocoel of each larva in each group. The time survival trend of each group post-infection was monitored at 25 °C and analyzed to estimate an LT_50_ as a virulence index. Moreover, total activities (U/mL) of ECEs and Pr1 proteases required for successful NCI [41] were quantified from the supernatants of 3-day-old liquid cultures, which were prepared by the shaking (150 rpm) incubation of a 10^4^ conidia/mL suspension in CDB-BSA (CDB amended with the sole nitrogen source of 0.3% bovine serum albumin (BSA)) at 25 °C [32,42]. Biomass levels in the cultures were assessed after vacuum drying. Hemolymph samples taken from surviving larvae were microscopically examined to show the status of fungal hemocoel colonization after CBI or NCI. CBI-killed larvae were held at 25 °C to show whether intrahemocoel hyphae penetrate through cuticle for outgrowth.

### 4.4. Subcellular Localization of Cla4A

The subcellular localization of Cla4A in responses to stress cues was shown with LSCM images of Cla4A-GFP expressed in a transgenic strain, which was generated by transforming the vector pAN52-cla4A-gfp-bar into WT. The transgenic strain’s conidia were suspended in SDBY (agar-free SDAY) for 60 h of shaking incubation at 25 °C, followed by 2 h of incubation in CDB alone or supplemented with chemical stressors (Figure 1B). Hyphae were stained with the nuclear dye DAPI and visualized for the green (expressed) and red (stained) fluorescence signals at the excitation/emission wavelengths of 358/460 and 488/507 nm, respectively.

### 4.5. Transcriptomic Analysis

Three 3-day-old SDAY cultures (replicates) of ∆*cla4A* and WT initiated with the spreading method at 25 °C and 12:12 (L–D) were sent to Lianchuan BioTech (Hangzhou, China) for transcriptomic analysis. All clean tags from RNA-seq datasets were mapped to the *B. bassiana* genome [43]. Both 1 ≤ log_2_ ratio ≤ −1 and FDR < 0.05 were significant levels used to identify DEGs, which were enriched to function categories and pathways via GO (https://www.geneontology.org) (accessed on 6 April 2024) and KEGG (https://www.genome.jp/kegg) (accessed on 6 April 2024) analyses at *p* < 0.05.

### 4.6. EMSAs for DNA-Binding Activity of Cla4A

Promoter DNA-binding elements of 130 DEGs across pathways were predicted as targets of Cla4A domains (residues 100–209, 212–247, and/or 562–830) at https://meme-suite.org/meme/tools/fimo/ (accessed on 6 April 2024). The product of *cla4A* was purified from the cell lysates of *Escherichia coli* via affinity chromatography and dialysis. EMSAs were performed to detect the binding activity of purified Cla4A extract (400 μg/mL) to the promoter DNAs of 12 Cla4A-targeting genes (Figure 6) amplified from the WT DNA with primers (Table S1). The promoter DNA (with no Cla4A-targeting site) of *cnB*, a coding gene of calcineurin subunit B (CnB) not dysregulated in the absence of *cla4A*, was used as a negative control. In each EMSA, 0.4 μg DNA samples were mixed with 1, 2, 3, 4, and 5 μg samples of Cla4A extract in 10 μL aliquots of binding buffer (25 mM HEPES (pH 7.4), 50 mM KCl, 5 mM MgCl_2_, 0.5 mM EDTA, 1 mM dithiothreitol, and 5% glycerol). After 30 min of reaction, Cla4A-bound DNA fragments were detected via agarose gel electrophoresis. The internal negative control was binding buffer containing 0.4 μg DNA or 5 μg protein sample alone.

### 4.7. Yeast Two-Hybrid (Y2H) Assays

Y2H assays were conducted to reveal whether Cla4A interacts with Ste11, Ste20, Cdc24, or Cdc42 in the MAPK/Fus3 cascade, like the Cla4-Cdc42 interaction documented in the yeast [7,14], and with Fpr3 in the C-C pathway since their coding genes were targets of Cla4A. The ORFs of *ste11* (BBA_02280), *ste20* (BBA_07438), *cdc24* (BBA_08316), *cdc42* (BBA_01874), and *fpr3* (BBA_00169) were cloned from the WT cDNA with primers (Table S1) and individually ligated to AD (prey vector pGADT7) and BD (bait vector pGBKT7), respectively. The two vectors verified by sequencing were transformed into the *S. cerevisiae* Y187 and Y2HGold strains, respectively, for 24 h of pairwise yeast mating at 30 °C on YPD (1% yeast extract, 2% peptone, 2% glucose plus 0.04% adenine hemisulfate salt). The yeast diploids were screened on double-dropout (SDM/-Leu/-Trp/X-α-Gal/AbA) and quadruple-dropout (SDM/-Leu/-Trp/-Ade/-His/X-α-Gal/AbA) plates. One positive control (AD-LargeT-BD-P53) and multiple negative controls (i.e., empty AD-BD and semi-empty constructs) were included in each assay. The yeast colonies on each plate were initiated by spotting 1 μL aliquots of 10^5^, 10^6^, and 10^7^ cells/mL suspensions and photographed after a 3-day incubation at 30 °C.

### 4.8. qPCR Analysis

Deletion mutants and control strains were incubated at 25 °C and 12:12 (L:D) for 3–6 days on SDAY plates inoculated with the spreading method. Total RNAs were extracted daily from the cultures using RNAiso Plus Kit (TaKaRa, Dalian, China) and reversely transcribed into cDNAs using PrimeScript RT reagent kit (TaKaRa). The cDNA samples were used as templates in qPCR analysis with primers (Tables S1) to quantify target gene transcripts under the action of SYBR Premix *Ex Taq* (TaKaRa), including the β-actin gene as a reference. A threshold cycle (2^−ΔΔCt^) method was used to assess relative transcript levels of *cla4A* and *cla4B* in the cDNA samples to verify expected recombination events in the mutants and of *brlA*, *abaA,* and *wetA* in the 3- to 6-day-old cultures of *cla4A* mutants versus WT.

### 4.9. Statistical Analysis

All phenotypic data from the experiments of three independent replicates were expressed as means plus standard deviations (SDs) and subjected to one-factor analysis of variance (ANOVA), followed by multiple comparisons of means among the tested strains via Tukey’s honestly significant difference (HSD) test.

## Data Availability

All experimental data are included in this paper and the Supplementary Material. All RNA-seq data from this study are available at the NCBI’s Gene Expression Omnibus under the accession no. PRJNA1048125 (https://www.ncbi.nlm.nih.gov/bioproject/1048125 (accessed on 6 April 2024)).

## Supplementary Materials

The following supporting information can be downloaded at: https://www.mdpi.com/article/10.3390/ijms25126410/s1.
