# Supplementary material for: Cla4A, a Novel Regulator of Gene Expression Networks Required for Asexual and Insect-Pathogenic Lifecycles of Beauveria bassiana
Source: Int J Mol Sci. 2024 Jun 10;25(12):6410. doi: 10.3390/ijms25126410 (PMC11203800; doi:10.3390/ijms25126410)
Supplement: Supplementary file 1 [file ijms-25-06410-s001.zip › Supporting Information File 1 (Figures S1-S3 and Table S1).pdf]

**Supporting Information File S1**

# Cla4A, a Novel Regulator of Gene Expression Networks Required for Asexual and Insect-Pathogenic Lifecycles of *Beauveria bassiana*

Si-Yuan Xu †, Rehab Abdelmonem Mohamed †, Lei Yu †, Sheng-Hua Ying and Ming-Guang Feng*

Institute of Microbiology, College of Life Sciences, Zhejiang University, Hangzhou, Zhejiang, 310058, China

† These authors contribute equally to this study. The author order is determined by the order of increasing seniority. * Corresponding author. *E-mail address*: mgfeng@zju.edu.cn (M.-G. Feng)

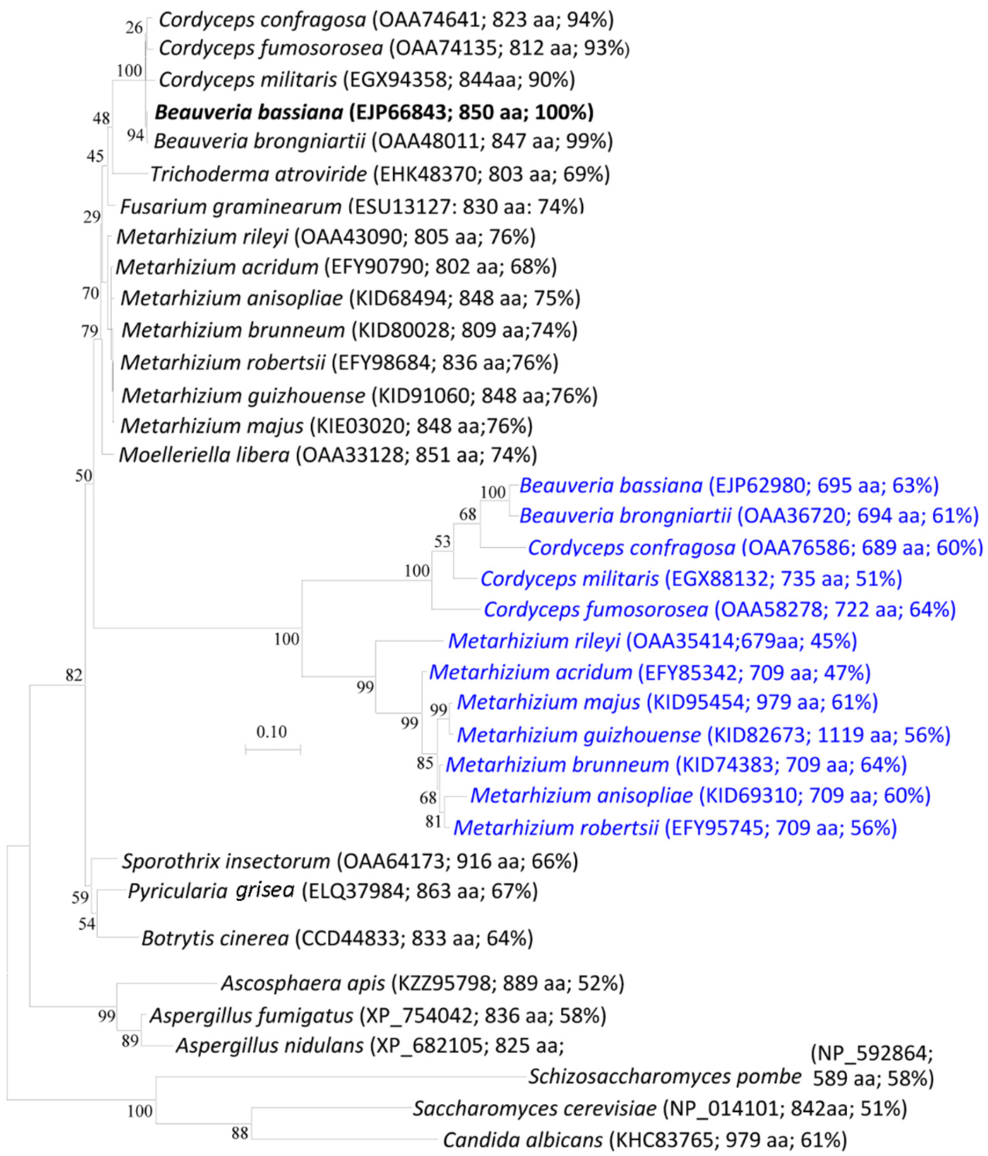


**Figure S1.** Phylogenetic tree of Cla4 homologs found in representative lineages of entomopathogenic and non-entomopathogenic ascomycetes. The tree was constructed with the maximum likelihood method in MEGA11 (http://www.megasoftware.net/). Bootstrap values of 1000 replications are shown at nodes. Scale bar: branch length proportional to genetic distance. The NCBI accession code and anomic acid sequence length of each homolog and its sequence identity to *B. bassiana* Cla4A (in bold) are given in the parentheses following the name of each fungus. Note that Cla4A and Cla4B (middle cluster in blue) exist only in the *Beauveria*/*Cordyceps* and *Metarhizium* lineages.

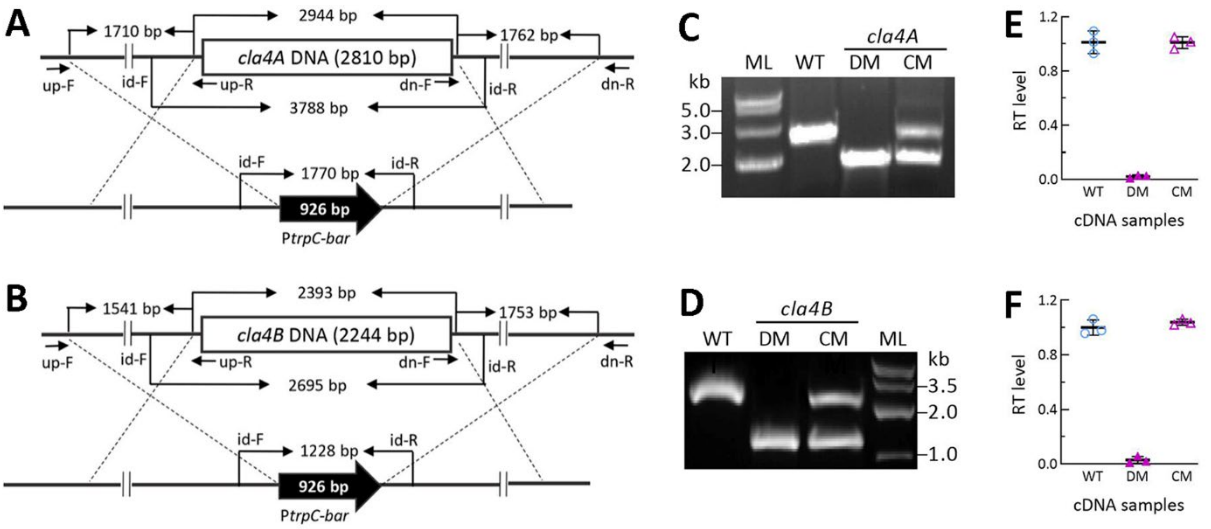


**Figure S2.** Generation and identification of *cla4A* and *cla4B* mutants in *B. bassiana*. (**A, B**) Schematic diagrams for deletion strategies of *cla4A* and *cla4B*. (**C, D**) The *cla4A* and *cla4B* mutants identified via PCR analysis. The DNA fragments detected in wild-type (WT), deletion mutant (DM) and complementation mutant (CM) indicate that a DNA fragment comprising partial flanking and full-length coding regions of each target gene was deleted in the DM strain as expected (3788 + 926 – 1770 = 2944 bp for *cla4A* deletion; 2695 + 926 – 1228 = 2393 bp for *cla4B* deletion). ML, molecular ladder of genomic DNA. (**E, F**) Relative transcript (RT) levels of *cla4A* and *cla4B* in the cDNA samples of their mutants with respect to the WT standard. Note that either *cla4A* or *cla4B* expression was abolished in the DM strain but restored to the WT level in the CM strain. Error bars: standard deviations of the means from three cDNA samples derived from independent 3-day-old SDAY cultures of each strain grown at an optimal regime.

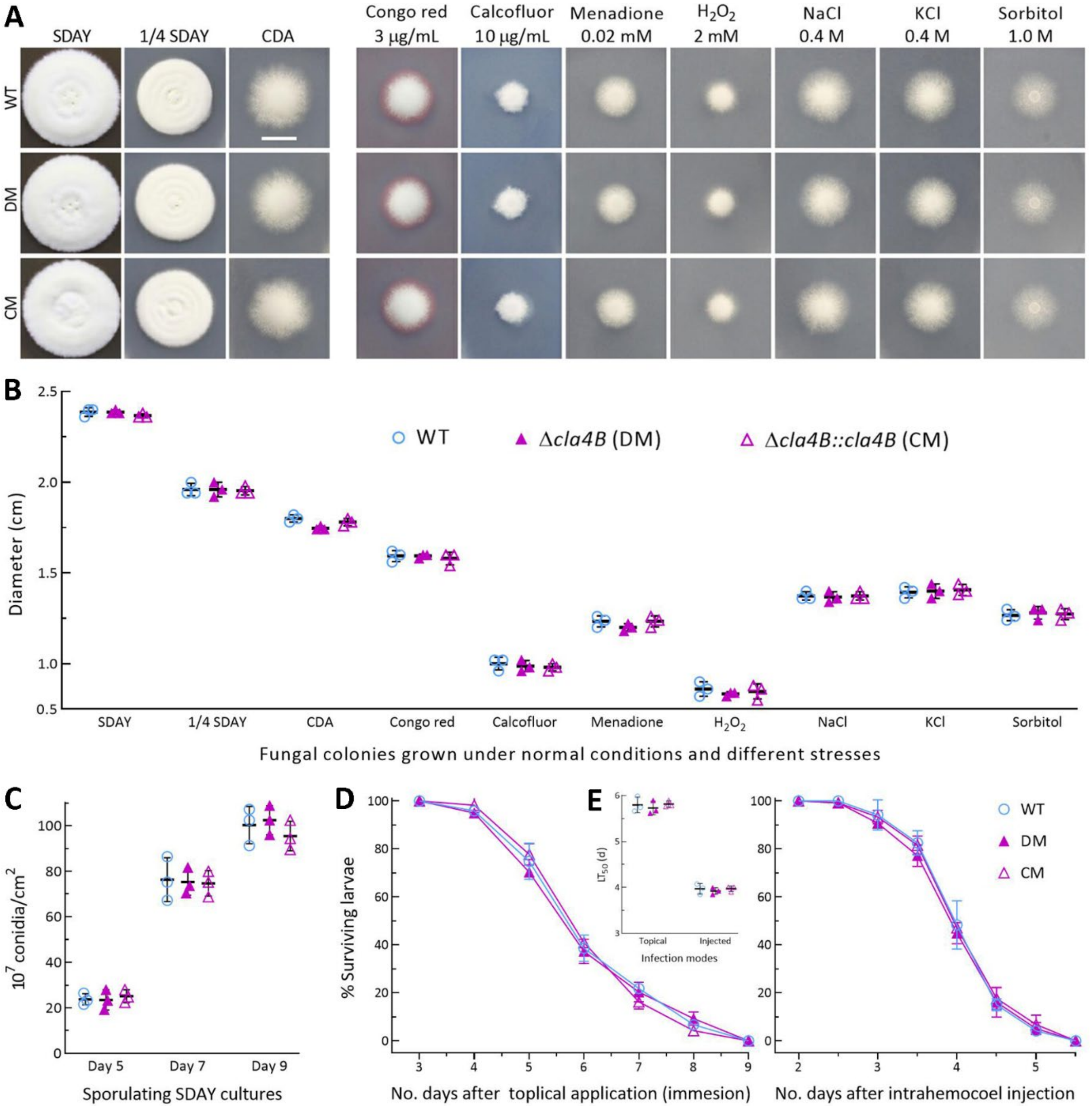


**Figure S3.** Dispensable role of Cla4B in the lifecycles *in vitro* and *in vivo* of *B. bassiana*. (**A, B**) Images and diameters of fungal colonies incubated 7 days at the optimal regime of 25°C and 12:12 (L:D) after initiation of colony growth by spotting 1 µL aliquots of a $10^6$ conidia/mL suspension on the plates of rich media (SDAY and 1/4 SDAY) and the minimal medium CDA alone or supplemented with indicated concentrations of chemical stressors. (**C**) Conidial yields assessed from the -5, 7- and 9-day-old SDAY cultures of tested strains, which were initiated by spreading 1 µL of a $10^7$ conidia/mL suspension per plate at the optimal regime. (**D, E**) Percent survival trends of *Galleria mellonella* larvae over the days after inoculation by topical application (immersion) of a $10^7$ conidia/mL suspension or intrahemocoel injection of ~500 conidia per larva and median lethal time ($LT_{50}$) estimates made by modeling analysis of the trends. Note that none of examined phenotypes differed significantly among the tested strains. Error bars: standard deviations of the means from three independent replicates.

**Table S1.** Paired primers used for manipulation and detection of targeted genes in *B. bassiana*.

| **Primers** | **Paired sequences (5′–3′)*** | **Purpose** |
|---|---|---|
| Cla4A-F/R | ACGAGCTGTACAAGTAA**CCCGGG**ATGTCACAGAACAGCGTATACTC / TGGCTGCAGGTCGAC**GGATCC**TTATTTGGCATGCTTCTTGAA | Cloning *cla4A* cDNA (2553 bp) for fusion to *GFP* |
| upCla4A-F/R | ACGAGCTGTACAAGTAA**CCCGGG**AGGGCATTGATGGTTCGT / TGGCTGCAGGTCGAC**GGATCC**AATCGGAAAGGCTCGTCA | Cloning *cla4A* 5′ fragment (1710 bp) for recombination/deletion |
| dnCla4A-F/R | GACCCATGGCTCGAG**TCTAGA**AAGGGCGGATACACGAC / GGTGGTGGTGGCTAGC**GTTAAC**AGAAAGGACGAGGCTGTG | Cloning *cla4A* 3′ fragment (1762 bp) for recombination/deletion |
| flCla4A-F/R | ATCCGTCGACCTGCAGCC**AAGCTT**AGGGCATTGATGGTTCGT / ACACTAGTCAGATCTTC**TAGTGT**CGGATGACGCAACAACG | Cloning full-length *cla4A* sequence for complementation (4990 bp) |
| upCla4B-F/R | ACGAGCTGTACAAGTAA**CCCGGG**AACGCCTTGGTGTTTGCC / TGGCTGCAGGTCGAC**GGATCC**TTGAAGCCGCCTTTGAGC | Cloning *cla4B* 5′ fragment (1541 bp) for recombination/deletion |
| dnCla4B-F/R | GACCCATGGCTCGAG**TCTAGA**TTGCGTAGGCTGTTCTCGG / GGTGGTGGTGGCTAGC**GTTAAC**TCGCCGTCTTCAAATCCAC | Cloning *cla4B* 3′ fragment (1753 bp) for recombination/deletion |
| flCla4B-F/R | ATCCGTCGACCTGCAGCC**AAGCTT**AGGTATTCTGGCGGGTCC / ACACTAGTCAGATCTTC**TAGTGT**CAAATGGGTCCGTAAAGAAAA | Cloning full-length *cla4B* sequence for complementation (4786 bp) |
| pCla4A-F/R | TCTATTTCCTCCATCTCCTTCC / ATCGTTCAATCGTCGCAAT | PCR detecting *cla4A* |
| pCla4B-F/R | GCAAATAAAACCTGCTAATGTCG / CCAAGGTCTACTCGGAAAACAAA | PCR detecting *cla4B* |
| qCla4A-F/R | CCCACCGACAGACATAGGCT / GCCCAGCCTTCCTTCTTGAT | qPCR detecting *cla4A* |
| qCla4B-F/R | AGAAGTCGCGTCCCTGAAAG / CAACTTCTTTGGTGCCGGTG | qPCR detecting *cla4B* |
| qBrlA-F/R | CGGCCGTTACTACATCCAGG / ACTCCGTTTCCATCGCACTT | qPCR detecting *brlA* |
| qAbaA-F/R | CCACGGCATGAACCTGTTTG / CGTTCGAGGGCAAAAAGTGG | qPCR detecting *abaA* |
| qWetA-F/R | AAGCCGGTGGGTTTACACTT / TTTGTCTTGGAGCTTCCGCT | qPCR detecting *wetA* |
| qActin-F/R | GGCAACATTGTCATGTCTGG / TTTGCTGGAAGGTGGATAGG | qPCR detecting β-actin gene |
| Pcdc24-F/R | CGCAACGCAGTCTTTTACA / CTTGGTCAAGCCTCCGAC | Cloning *cdc24* promoter (1590 bp) |
| Pcdc42-F/R | ACCGAGGGAGGAGCTGTTC / CGGCCATGGTTCAGATCTG | Cloning *cdc42* promoter (1627 bp) |
| Pste11-F/R | AAATAGGTGATGTCTGATAATGGC / TGGTGGCGGTTGCGTCT | Cloning *ste11* promoter (1633 bp) |
| Pste20-F/R | TGCTGGTAAATAGTGATGAACAAGTT / TTGTCCATGTTGGGGGGG | Cloning *ste20* promoter (1610 bp) |
| Pbck1-F/R | TACGATATGGACGATAAGAAGGC / CATGTTGGGCGACGTTTG | Cloning *bck1* promoter (1698 bp) |
| Psmp1-F/R | TGTTGGTGAATGAGTCGGATA / ACCCATGGTGACTGGATTGTA | Cloning *smp1* promoter (1578 bp) |
| PsskB-F/R | AAGCATCGCTACCTCGTGG / GCACGGTTGTCCATTGTCTT | Cloning *sskB* promoter (1644 bp) |
| Ppbs2-F/R | CTGGCATTATCCCGACTGTG / CATCGAGGTTTCGAGTTCTTTT | Cloning *pbs2* promoter (1678 bp) |
| Pmsn2-F/R | GACGGCTTCCTACTCCAACC / TCCATTGTGAGTGATTGTTTCG | Cloning *msn2* promoter (1584 bp) |
| Pfpr3-F/R | GAGCACAGCCACTTCAGACC / CAGCCATTTTGAAGATGGAGAT | Cloning *fpr3* promoter (1810 bp) |
| Pcrz1-F/R | CCTAACCCTACTGATAACTCCGA / GTCGAGAAGATGAGAGAAAGCG | Cloning *crz1* promoter (1629 bp) |
| Pvcx3-F/R | GGCGGGCTATCTGGAGTAAA / GGTGGCCAGCCATACGCT | Cloning *vx3* promoter (2123 bp) |
| Pphr2-F/R | ATACTGGGGACAGACACTTGC / GTTTCCTGATTCGAAACTGGA | Cloning *phr2* promoter (1904 bp) |
| PcnB-F/R | AAGGGGACTGGGTAAAGCTG / CGGAGAGCGAGCGACAG | Cloning *cnB* promoter (1571 bp) |
| bdCla4A-F/R | ATGGCCATGGAGGCC**GAATTC**ATGTCACAGAACAGCGTATA / CGCTGCAGGTCGAC**GGATCC**TTATTTGGCATGCTTCTTGA | Cloning *cla4A* cDNA (2550 bp) for ligation to BD |
| adCdc24-F/R | GCCATGGAGGCCAGT**GAATTC**ATGAAGAGCGCCCCTCGAAG / CAGCTCGAGCTCGAT**GGATCC**CTAGGAAACGTCACCCACGC | Cloning *cdc24* cDNA (2913 bp) for ligation to AD |
| adCdc42-F/R | GCCATGGAGGCCAGT**GAATTC**ATGGCCGTCGTCGCAACCAT / CAGCTCGAGCTCGAT**GGATCC**TTACAAAATGCGACATTTGT | Cloning *cdc42* cDNA (585 bp) for ligation to AD |
| adSte11-F/R | GCCATGGAGGCCAGT**GAATTC**ATGCTGGCCACCAAGCCGCC / CAGCTCGAGCTCGAT**GGATCC**TCACGTGATGGGGTTGAGAA | Cloning *ste11* cDNA (2727 bp) for ligation to AD |
| adSte20-F/R | GCCATGGAGGCCAGT**GAATTC**ATGGACAAGAATTCGTCCCA / CAGCTCGAGCTCGAT**GGATCC**TCACGCTTTGCGCAACTTTT | Cloning *ste20* cDNA (2640 bp) for ligation to AD |
| adFpr3-F/R | GCCATGGAGGCCAGT**GAATTC**ATGGCTGGCATCTCTGGTCC / CAGCTCGAGCTCGAT**GGATCC**CTTGATCTCGAGCAGCTTGA | Cloning *fpr3* cDNA (1389 bp) for ligation to AD |

* Underlined regions are DNA fragments to exchange for the corresponding fragments of vectors constructed at the sites (in bold) of restriction enzymes for *cla4A* fusion to N-terminus of *GFP* (*Bam*HI/*Xma*I), homogenous recombination of *bar*-separated 5′ and 3′ fragments for *cla4A* or *cla4B* deletion (*Bam*HI/*Xma*I and *Xba*I/*Hpa*I), gateway exchange fragment for targeted gene complementation (*Hin*dIV/*Xba*I), or ligation of targeted gene to BD or AD (*EcoR*I/*BamH*I) for Y2H assays.
